# Prenatal exposure to fine particulate matter and the risk of spontaneous preterm birth: A population-based cohort study of twins

**DOI:** 10.3389/fpubh.2022.1002824

**Published:** 2022-10-24

**Authors:** Ping Qiao, Kechen Fan, Yirong Bao, Ling Yuan, Haidong Kan, Yan Zhao, Jing Cai, Hao Ying

**Affiliations:** ^1^Departments of Obstetrics, Shanghai First Maternity and Infant Hospital, Tongji University School of Medicine, Shanghai, China; ^2^Shanghai First Maternity and Infant Hospital, Tongji University School of Medicine, Shanghai, China; ^3^Key Lab of Public Health Safety of the Ministry of Education and National Health Commission Key Laboratory of Health Technology Assessment, School of Public Health, Fudan University, Shanghai, China; ^4^Shanghai Key Laboratory of Maternal Fetal Medicine, Shanghai, China

**Keywords:** ambient air pollution, PM_2.5_, spontaneous preterm birth, preterm birth, twin pregnancy

## Abstract

**Background:**

Studies in singletons have suggested that prenatal exposure to fine particulate matter (PM_2.5_) and some of its chemical components is associated with an increased risk of preterm birth (PTB). However, no study has been conducted in twins.

**Purpose:**

To examine the associations of maternal exposure to total PM_2.5_ mass and its carbonaceous components with PTB in twin pregnancies.

**Methods:**

A total of 1,515 pairs of twins and their mothers were enrolled from a previous twin birth cohort that had been conducted at the Shanghai First Maternity and Infant Hospital School of Medicine of Tongji University in China. Participants who had iatrogenic PTBs were excluded. Maternal exposure to total PM_2.5_ mass and two carbonaceous components, namely, organic carbon (OC) and black carbon (BC), was estimated by a satellite-based model. The associations between PM_2.5_ exposure and the risk of spontaneous PTB were evaluated by logistic regression analysis.

**Results:**

This study found that exposure to total PM_2.5_ mass and OC during the second trimester of pregnancy was significantly associated with an increased risk of spontaneous PTB. An interquartile range (IQR) increase in total PM_2.5_ mass and OC exposure during the second trimester was associated with 48% (OR = 1.48, 95% CI, 1.06, 2.05) and 50% (OR = 1.50, 95% CI, 1.00, 2.25) increases in the odds of PTB, respectively. However, no significant association was found between BC exposure during any exposure window and the risk of PTB.

**Conclusion:**

The findings suggest that exposure to ambient air pollution with fine particles may be a risk factor for spontaneous PTB in twin pregnancies. The middle stage of pregnancy seems to be a critical window for the impacts of PM_2.5_ exposure on PTB in twin pregnancies.

## Introduction

With the extensive development of assisted reproductive technology (ART) and delayed childbearing age, the incidence of twin pregnancy has dramatically increased worldwide (1–3). The prevalence of twin pregnancy has been reported to be 3.26% in the U.S. population (4). The latest statistics involving 556,298 births from 64 different levels of Chinese medical care showed that the incidence of twin pregnancy was 3.69% in the Chinese population, showing an upward trend (5). Twin pregnancies have all the complications of singleton pregnancies but at higher rates, especially preterm birth (PTB). The incidence of PTB among twin pregnancies is as high as 60%, which is more than 6–10 times higher than that among singleton pregnancies (4).

PTB is a major public health challenge, with its complications accounting for an estimated 35% of 3.1 million annual neonatal deaths worldwide (6). It not only influences early-life outcomes but is also associated with an increased risk of cerebral palsy, long-term neurodevelopmental impairment, and metabolic and cardiovascular diseases, which places a heavy financial and spiritual burden on affected families as well as societies (7). It is therefore crucial to identify potential modifiable risk factors, which is of great importance to public health.

Fine particulate matter (PM_2.5_), which refers to particulate matter that is <2.5 μm in aerodynamic diameter, is the most serious environmental threat worldwide. It is a complex mixture comprising various components. Several studies in singleton pregnancies have shown that prenatal exposure to PM_2.5_ and some of its chemical components, such as carbonaceous species (8, 9), is responsible for the increased risk of PTB (10–13). However, the effect of PM_2.5_ exposure on spontaneous PTBs in twin pregnancies remains unknown.

It is more challenging to investigate the effects of PM_2.5_ on multiple preterm births because the etiology is more complex and varied. Furthermore, because of the inadequate sample size, insights that are specific to multiple gestations cannot be obtained. Tracking “medically indicated” (defined as an elective cesarean section or induced labor before 37 weeks of gestation due to maternal complications or fetal indications, which is also called “iatrogenic”) (14) vs. “non-indicated” provider-initiated PTBs would be crucial, but definitions and data are missing, and this distinction is not readily available at the national level or consistently over time, even in developed countries.

Therefore, the purpose of this study was to investigate the associations between maternal exposure to PM_2.5_ and its carbonaceous components and spontaneous PTB in a twin birth cohort in Shanghai, China, by maximizing the control for a variety of potential confounders.

## Methods

### Study population

Participants in the present study were from a previous twin birth cohort that had been conducted at the Shanghai First Maternity and Infant Hospital School of Medicine of Tongji University in China. The recruitment and eligibility criteria have been described in detail and are as follows. Briefly, twin pregnancies delivered after 24 weeks of gestation and with two liveborn infants were included in the cohort. The original cohort excluded twin pregnancies with genetic or structural abnormalities of either fetus, monochorionic twin pregnancies complicated by twin-twin transfusion syndrome (TTTS) or twin anemia-polycythemia sequence (TAPS), pregnancies with multifetal pregnancy reduction (MFPR), and pregnancies with a known history of chronic hypertension, diabetes, and immunological or kidney disease before the pregnancy. A total of 2,122 twin pregnancies were included in the cohort between January 2013 and June 2016. In the present study, participants who had iatrogenic PTBs (*N* = 402) and who were not locally living in the city of Shanghai (*N* = 205) were excluded. This resulted in a final analysis of 1,515 pairs of twins and their mothers. The study protocols were approved by the Ethics Committee of the Shanghai First Maternity and Infant Hospital.

### Data collection and outcome definitions

Data on residential addresses, maternal age (MA), prepregnancy height and weight, gravidity and parity, and the use of ART were collected during each participant's first prenatal visit (before 16 weeks of gestation). Data on twin chorionicity, intrauterine treatment, pregnancy complications, such as pregnancy-associated hypertensive disorders, gestational diabetes mellitus (GDM), TTTS, and selective fetal growth restriction (sFGR), were obtained from obstetric records, which were completed by midwives and obstetricians. Twin birth outcomes, including the gestational age (GA), mode of delivery, Apgar score, birth weight, birth length, and sex, were abstracted from newborn discharge records. The outcome of interest in the present study was PTB, which was generally defined as a delivery occurring before 37 weeks of gestation. GA was calculated based on a pregnant woman's last menstrual period (LMP) and was confirmed or corrected by ultrasound measurement of the crown-rump length (CRL) in the first trimester of pregnancy. Chorionicity was confirmed by determining the number of gestational sacs between 6 + 0 and 9 + 0 weeks and the lambda and T signs between 11 + 0 and 13 + 6 weeks of gestation (15).

### PM_2.5_ exposure assessment

Individual exposure to total PM_2.5_ mass and its 2 carbonaceous constituents during pregnancy were assessed based on each participant's residential address. The 2 carbonaceous constituents were organic carbon (OC) and black carbon (BC), which have been associated with an increased risk of PTB in singleton pregnancies. Data were calculated for four exposure windows for each participant: the entire period of pregnancy, first trimester (0–90 days) of pregnancy, second trimester (91–180 days) of pregnancy and third trimester of pregnancy (day 181 of pregnancy to delivery) based on monthly concentrations of PM_2.5_.

The concentrations of total PM_2.5_ mass and its chemical constituents during the study period were derived from a satellite-based model. The exposure assessment model was from the V4.CH.02 product of the Atmospheric Composition Analysis Group (ACAG), which extends the approach of van Donkelaar et al. (16) to provide ground-level predictions of total PM_2.5_ and its main chemical constituents, including over China. Specifically, this model combines multiple satellite products of aerosol optical depth (AOD) retrievals and determines ground-level PM_2.5_ concentrations based on total mass and the simulated geophysical relationship between PM_2.5_ and AOD. The model for China was built using ground monitoring data from ~1,000 monitors at a monthly timescale with a 1 × 1 km resolution, resulting in an overall R^2^ of 0.78, vs. using cross-validated ground-based monitors over China. GEOS-Chem simulation was applied to partition the total PM_2.5_ mass into several compositions.

The time resolution of the PM_2.5_ exposure data was monthly. If a pair of twins was born in the middle of 1 month, their trimester-specific exposures were calculated according to a time-weighted method. Briefly, if the conception date was on day Dj of month Mi, the first month of pregnancy ended 30 days later, which was approximately Dj of month Mi+1. Then, the concentration of the first month (Con. 1st month) was calculated according to the following equation:


$$\begin{array}{l} \text{Con}.\;\left(1^{\text{st}}\text{ month}\right)=\frac{\left(30-\;D_{j}\right)/30\;\times \;Con.\;M_{i}+\;D_{j}\;/30\times \text{ Con}.\;M_{i+1}}{2} \end{array}$$

The first trimester covered the first 3 months (90 days) of pregnancy, and the concentrations of the first trimester were calculated as the mean exposure of the first 3 months. Then, the exposure levels of the second and third trimesters were calculated accordingly.

### Statistical analysis

The general characteristics of the participants are presented as percentages (%) and were compared between PTB cases and term birth control cases using the χ^2^ test. The PM_2.5_ exposure levels were not normally distributed, and medians (25th, 75th percentile) are presented to characterize their distribution. The potential differences in PM_2.5_ exposure levels between PTB cases and term birth control cases were compared by means of the Mann–Whitney *U*-test.

The associations between PM_2.5_ exposure and the risk of PTB were evaluated by logistic regression analysis using odds ratios (ORs) as the risk measure, with 95% confidence intervals (CIs). MA, parity, prepregnancy body mass index (BMI), gestational hypertension disorders, GDM, the use of ART, twin chorionicity, twin growth discordance and birth seasons were adjusted in the logistic regression models. These covariates were included because of data accessibility and their associations with the exposure or outcomes. To further explore the exposure-response relationships between PM_2.5_ exposure and the risk of PTB, exposure levels to total PM_2.5_ mass or each chemical constituent were categorized into quartiles and estimated ORs for each quartile with the first quartile as a reference. The *P*-value for trend was estimated by including the categorized exposure data as continuous variables.

Sensitivity analyses were subsequently conducted to examine the impacts of gestational hypertensive disorders and GDM on the associations between PM_2.5_ exposure and PTB. All logistic regression analyses were replicated and restricted to participants without gestational hypertension disorders or GDM. All statistical analyses were performed using SPSS 16.0 (SPSS, Chicago, IL), and a two-sided *P* < 0.05 was considered statistically significant.

## Results

### Population characteristics

Table 1 presents the general characteristics of the participants. The majority of the participating women were nulliparous (81.5%), aged <35 years (78.1%), and conceived naturally (73.3%). Among the 1,515 pairs of twins, the incidence of PTB was 50.4% (764/1,515). The PTB group and term birth group differed in the use of ART, twin chorionicity and the incidence of gestational hypertension. Results of the χ^2^ test showed that there were no significant differences in MA (*P* = 0.205), parity (*P* = 0.812), prepregnancy BMI (*P* = 0.080), the season of delivery (*P* = 0.653), or the incidence of GDM (*P* = 0.502) between the two groups.

**Table 1:** General characteristics of the study population.

**Characteristics**	**Total, *n* (%)**	**PTB, *n* (%)**	**Non-PTB, *n* (%)**	***p*** **-value^a^**
	***N*** **= 1,515**	***N*** **= 764**	***N*** **= 751**	
**Maternal age (years)**				
<35	1,183 (78.1)	607 (79.5)	576 (76.7)	0.205
≥35	332 (21.9)	157 (20.5)	175 (23.3)	
**Prepregnancy BMI (kg/m** ^**2**^ **)**				
<18.5	92 (6.1)	50 (6.5)	42 (5.6)	0.080
18.5–24.9	1,028 (67.9)	498 (65.2)	530 (70.6)	
≥25	395 (26.1)	216 (28.3)	179 (23.8)	
**The use of ART**				
Yes	404 (26.7)	179 (23.4)	225 (30.0)	0.004^**^
No	1,111 (73.3)	585 (76.6)	526 (70.0)	
**Parity**				
Nulliparous	1,235 (81.5)	621 (81.3)	614 (81.8)	0.812
Multiparous	280 (18.5)	143 (18.7)	137 (18.2)	
**Twin chorionicity**				
Dichorionic	1,174 (77.5)	473 (61.9)	701 (93.3)	0.000^**^
Monochorionic	341 (22.5)	291 (38.1)	50 (6.7)	
**Season of delivery** ^a^				
Spring	498 (32.9)	262 (34.3)	236 (31.4)	0.653
Summer	338 (22.3)	166 (21.7)	172 (22.9)	
Autumn	265 (17.5)	134 (17.5)	131 (17.4)	
Winter	414 (27.3)	202 (26.4)	212 (28.2)	
**GDM**				
Yes	344 (22.7)	168 (22.0)	176 (23.4)	0.502
No	1,171 (77.3)	596 (78.0)	575 (76.6)	
**Gestational hypertension**				
Yes	268 (17.7)	212 (27.7)	56 (7.5)	0.000^**^
No	1,247 (82.3)	552 (72.3)	695 (92.5)	

a p-values were calculated by the χ^2^-test.

** p < 0.01.

### PM_2.5_ exposure levels

The distributions of exposure levels to total PM_2.5_ mass and its 2 carbonaceous constituents, namely, OC and BC, during the whole pregnancy and each trimester are shown in Table 2. The exposure levels to total PM_2.5_ mass, OC and BC varied across pregnancy trimesters. During the whole pregnancy, the median exposure levels to total PM_2.5_ mass, OC and BC were 52.50, 9.23, and 3.59 μg/m^3^, respectively. The correlations between exposure to total PM_2.5_ mass, OC and BC for each trimester and the whole pregnancy are shown in Supplementary Table 1. The results showed that the exposure levels to total PM_2.5_ mass, OC and BC were highly correlated with each other, with correlation coefficients ranging from 0.83 to 0.96.

**Table 2:** Descriptive statistics of air pollution exposure levels (μg/m^3^).

	**Total**	**PTB**	**Non-PTB**	***p*** **-value^a^**
**PM**_**2.5**_				
Trimester 1	49.25 (42.00, 57.00)	48.75 (42.00, 56.75)	49.75 (42.00, 57.50)	0.388
Trimester 2	52.50 (44.50, 61.00)	53.50 (45.00, 61.75)	51.75 (44.00, 60.25)	0.013^*^
Trimester 3	53.00 (45.67, 61.25)	53.00 (45.67, 60.75)	53.25 (45.67, 61.50)	0.822
Entire pregnancy	52.50 (49.67, 54.56)	52.56 (49.56, 54.78)	52.40 (49.78, 54.33)	0.565
**OC**				
Trimester 1	5.70 (4.40, 12.25)	5.73 (4.40, 12.14)	5.67 (4.40, 12.42)	0.729
Trimester 2	8.27 (4.88, 14.05)	8.44 (4.91, 14.43)	8.05 (4.85, 13.67)	0.075
Trimester 3	8.28 (4.90, 14.22)	8.03 (4.90, 14.05)	8.40 (4.93, 14.27)	0.778
Entire pregnancy	9.23 (8.21, 9.98)	9.30 (8.11, 10.13)	9.18 (8.23, 9.88)	0.208
**BC**				
Trimester 1	3.15 (2.80, 4.00)	3.15 (2.80, 3.95)	3.15 (2.80, 4.05)	0.661
Trimester 2	3.52 (2.97, 4.35)	3.55 (3.00, 4.40)	3.50 (2.93, 4.30)	0.091
Trimester 3	3.45 (2.95, 4.33)	3.43 (2.97, 4.30)	3.52 (2.93, 4.33)	0.913
Entire pregnancy	3.59 (3.32, 3.76)	3.59 (3.29, 3.77)	3.58 (3.35, 3.74)	0.703

a p-values were calculated by the Mann–Whitney U-test.

* p < 0.05.

Analyses of potential differences in PM_2.5_ exposure levels between the PTB and term birth groups were conducted using the Mann–Whitney *U*-test. The results showed that total PM_2.5_ mass exposure during the second trimester was significantly higher in the PTB group than in the term birth group (53.50 vs. 51.75 μg/m^3^, respectively, *P* = 0.013). No significant difference was found for OC or BC exposure between the two groups (Table 2).

### Association between PM_2.5_ exposure and PTB risk

Table 3 shows the associations between total PM_2.5_ mass exposure, carbonaceous constituent exposure and the risk of PTB. Only total PM_2.5_ mass exposure during the second trimester was significantly associated with an increased risk of PTB. In the adjusted model, an interquartile range (IQR; 16.5 μg/m^3^) increase in total PM_2.5_ mass exposure during the second trimester was associated with a 48% (OR = 1.48, 95% CI, 1.06, 2.05) increase in the odds of PTB.

**Table 3:** Associations of total PM_2.5_ mass and exposure to two carbon constituents with the risk of preterm birth.

**Exposure time window**	**ORs (95 CI%) Adjusted model** ^a^		
	**PM_2.5_ total mass**	**OC**	**BC**
Trimester 1	0.84 (0.67, 1.06)	0.85 (0.64, 1.14)	0.83 (065, 1.07)
Trimester 2	1.48 (1.06, 2.05)^*^	1.50 (1.00, 2.25)^*^	1.40 (0.99, 2.00)
Trimester 3	0.79 (0.60, 1.05)	0.73 (0.52, 1.04)	0.73 (0.51, 1.04)
Entire pregnancy	0.95 (0.81, 1.11)	0.93 (0.79, 1.09)	0.94 (0.79, 1.13)

a Models adjusted for MA, prepregnancy BMI, gestational hypertension disorders, GDM, the use of ART, parity, twin chorionicity, twin growth discordance, and birth season.

* p < 0.05.

Similar to total PM_2.5_ mass exposure, OC exposure during the second trimester was found to be significantly associated with an increased risk of PTB. For an IQR (9.17 μg/m^3^) increase in OC exposure during the second trimester, the odds of PTB increased by 50% (OR = 1.50, 95% CI, 1.00, 2.25). However, no significant association was found between BC exposure during any exposure window and PTB.

**Figure 1:**
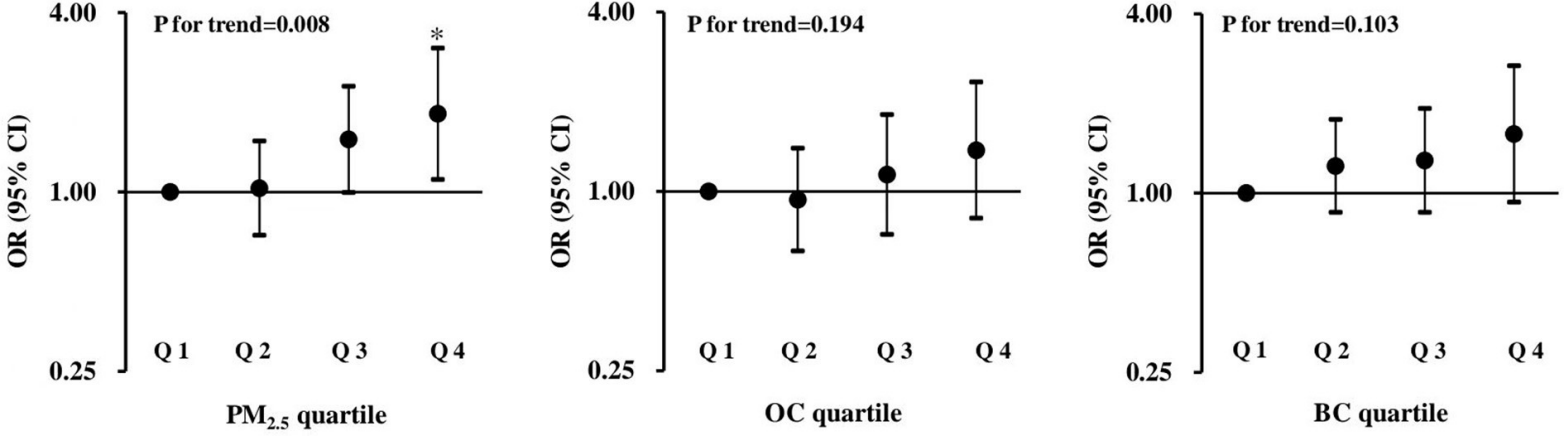
ORs and 95% CIs for the associations between total PM_2.5_ mass and two carbon constituents with preterm birth. The first quartile is the reference, and all models were adjusted for MA, prepregnancy BMI, gestational hypertension disorders, GDM, the use of ART, parity, twin chorionicity, twin growth discordance and the season of delivery. *p < 0.05.

Given that the PTB risk was significantly positively correlated with total PM_2.5_ mass or OC exposure during the second trimester of pregnancy, the exposure-response relationships of PM_2.5_ exposure with PTB risk were further explored by categorizing the distribution of total PM_2.5_ mass or each chemical constituent exposure into quartiles. As shown in Figure 1, significant positive associations with the risk of PTB were observed for total PM_2.5_ mass exposure, with a monotonic linear increase in ORs across quartiles (*P* for trend = 0.008).

### Sensitivity analyses

To exclude the impacts of gestational hypertensive disorders and GDM on the associations between PM_2.5_ exposure and PTB, the regression analysis was restricted to participating women without gestational hypertensive disorders or GDM. As shown in Supplementary Table 2, the associations of total PM_2.5_ mass and OC exposure during the second trimester with PTB were not appreciably changed.

## Discussion

In this study, the associations of maternal exposure to total PM_2.5_ mass and its carbonaceous components (OC and BC) with the risk of spontaneous PTB in twin pregnancies were examined. Exposure to total PM_2.5_ mass and OC during the second trimester of pregnancy was associated with an increased risk of PTB. The results suggest that maternal exposure to PM_2.5_ may be a risk factor contributing to PTB in twin pregnancies.

Most previous studies have reported significant associations between PM_2.5_ exposure and an increased risk of PTB in singletons, despite the heterogeneity in study designs and PM_2.5_ exposure assessment methods (17–20). For example, in a retrospective cohort study including 231,637 births, Basu et al. found a 16.4% increase in the odds of PTB per IQR increase in PM_2.5_ exposure during the whole pregnancy (9). In another study, using data from 183 individual countries, Mallry et al. estimated that the percentage of PTB attributed to PM_2.5_ exposure was 18% (21). The reasons for the increased incidence of PTB in twin pregnancies are multifactorial and include maternal complications, such as preeclampsia and intrahepatic cholestasis of pregnancy, and fetal complications, which are unique to twin pregnancies and directly related to chorionicity. Spontaneous PTB of multiple gestations may also have unique predisposing factors, including a short cervical length, uterine hypertonicity or distension, intrauterine infection or inflammation (22). Studies have shown that PM_2.5_ has the ability to activate multiple pathophysiological processes, including oxidative stress, DNA damage, immunological alteration and inflammation (23, 24). PM_2.5_ can potentially be deposited deep into the lungs and may enter the circulatory system, which may induce systemic inflammation, or enter the placenta through simple diffusion (25). The direct toxic effects of PM_2.5_ on placental inflammation (26) may potentially result in altered placental vascular function (27), triggering the occurrence of PTB. In addition, PM_2.5_ may affect placental vasoconstriction, interfering with placental oxygen and nutrient transport and leading to fetal hypoxia and PTB (28). A larger placental mass is associated with increased secretion of mediators, such as corticotrophin-releasing hormone (CRH), which is correlated with the onset of parturition (22). In the present study of spontaneous PTB, a 48% (OR = 1.48, 95% CI, 1.06, 2.05) increase in the odds of PTB was found to be associated with an IQR increase in total PM_2.5_ mass, and the estimated effect seemed to be larger than that in singletons. Possible reasons may be that a greater twin placental mass leads to increased placental inflammatory responses to PM_2.5_ and increased CRH secretion. Further studies are warranted to understand the biological mechanisms of PM_2.5_-induced PTB.

The observed positive associations of OC and PM_2.5_ exposure with PTB risk in twins were consistent with the data of a previous study conducted on singletons (29). Shanghai is a city with relatively serious organic carbon air pollution, and Xu et al. identified that biomass burning from the adjacent Yangtze River Delta region was responsible for the high OC plumes during the harvest season (30). Bové et al. reported that BC could pass through the placenta and accumulate on the fetal side of the placenta, which may represent a potential mechanism that leads to the occurrence of preterm birth (31). Previous studies conducted among singletons also reported significant associations between prenatal BC exposure and an increased risk of preterm birth (28, 32, 33). In this study, the associations of prenatal BC exposure with PTB risk were marginally significant, with an OR of 1.40 (95% CI, 0.99, 2.00). The magnitude and direction of the associations were generally similar to those of OC exposure with PTB. The lack of significant associations might be due to the lower exposure levels of BC (mean exposure level of BC, 3.59 μg/m^3^; mean exposure level of OC, 9.23 μg/m^3^) and the relatively small sample size of this study.

Identifying critical exposure windows would allow for the design of targeted prevention strategies, as well as the exploration of the potential biological mechanisms underlying the associations of PM_2.5_ exposure with PTB. In previous research evaluating the effect of gestational PM_2.5_ exposure on pregnancy complications of placental origin, the critical window was identified as the first trimester (34). In this study, PM_2.5_ exposure during the second trimester demonstrated significant associations with PTB, which is consistent with some previous epidemiological studies (35–37), whereas other studies identified the late stage of pregnancy as the most significant critical window (19, 20, 38). This window of susceptibility (the second trimester) was considered to be biologically plausible because Mayer's study showed that from the second trimester, fetal-placental blood flow starts to increase continuously with advancing gestation to meet the increasing fetal demand for oxygen and nutrients (39), which in turn leads to elevated fetal exposure to endogenous and exogenous factors such as an inhalational dose of ambient PM_2.5_ and maternal systemic inflammatory mediators (40).

### Strengths

This study extends previous epidemiological research on air pollution and PTB in singletons and suggests that maternal exposure to PM_2.5_ during the second trimester is an important risk factor for PTB in twin pregnancies. The strengths of the study included the relatively large sample size of twins and the ability to control for a variety of potential confounders, such as MA, prepregnancy BMI, the use of ART, parity, twin chorionicity, twin growth discordance and the season of delivery and maternal pregnancy complications. The use of administrative databases improved the ability to distinguish spontaneous from medically indicated births, which makes this study on PM_2.5_ and spontaneous PTB in twin pregnancies more scientific by excluding provider-initiated PTB for maternal or complex twin indications.

### Limitations

However, several limitations should be considered. First, all participants with twin pregnancies were recruited from a hospital-based birth cohort, which was subject to an inevitable selection bias and limited the generalizability of the results. Second, although comprehensive confounders were considered in the data analysis, some known influencing factors of PTB, such as maternal education level, occupation, sleep status during pregnancy, passive smoking, and socioeconomic and nutritional status, were unavailable in the analysis; thus, residual confounding was possible Third, the exposure assessment mainly relied upon exposure at the maternal residence and ignored the mobility of each participant between their residence and workplace, which may cause exposure misclassification. However, the study by Pereira et al. indicated that maternal residential mobility had only a slight or even no impact on the direction of the effect estimate (41). Fourth, information on possible treatments to prevent recurrent PTB, such as progesterone, pessary, or cerclage, was not available in the selected studies. Finally, this was only an observational study, and the biological mechanisms underlying the association of PM_2.5_ exposure with PTB need to be further investigated. Although we tried our best to comprehensively collect as many covariates as possible, future research with a larger sample size and more optimized research design is still needed to verify the phenomenon in the future.

## Conclusion

It was found that maternal exposure to total PM_2.5_ mass and OC during the second trimester of pregnancy was associated with an increased risk of PTB in twin pregnancies. These findings suggest that exposure to PM_2.5_ during the middle stage of pregnancy may have a role in the etiology of PTB. Further studies are necessary to confirm the findings.

## Data availability statement

The data analyzed in this study is subject to the following licenses/restrictions: the dataset generated during and/or analyzed during the current study are not publicly available due to ethics reason. Requests to access these datasets should be directed to zzybbmm@aliyun.com.

## Ethics statement

The study protocols were approved by the Ethics Committee of the Shanghai First Maternity and Infant Hospital (No. KS18118). This was a retrospective study. All data were extracted from the medical records and were anonymized, and no written consent was obtained from the participating pregnant women.

## Author contributions

PQ: conceptualization, investigation, and writing-original draft preparation. KF: software and formal analysis. YB and LY: data curation. HK: supervision. YZ: validation, software, and formal analysis. JC: methodology, software, and formal analysis. HY: conceptualization, project administration, supervision, and funding acquisition. All authors contributed to the article and approved the submitted version.

## Funding

We would like to express a deep gratitude for financial support from the National Key Research and Development Program (Nos. 2022YFC2704600 and 2022YFC2704604), the National Natural Science Foundation of China (Nos. 82071678, 82271719, 8217060048, and 8190061792), Clinical Research Plan of SHDC (SHDC2020CR2059B), Shanghai Municipal Science and Technology Commission Research Fund (No. 21140903800), and Key Research Project of Pudong New Area Population and Family Planning Commission (No. PW2020E-1).

## Conflict of interest

The authors declare that the research was conducted in the absence of any commercial or financial relationships that could be construed as a potential conflict of interest.

## Publisher's note

All claims expressed in this article are solely those of the authors and do not necessarily represent those of their affiliated organizations, or those of the publisher, the editors and the reviewers. Any product that may be evaluated in this article, or claim that may be made by its manufacturer, is not guaranteed or endorsed by the publisher.

## Abbreviations

PM_2.5_: fine particulate matter
PTB: preterm birth
OC: organic carbon
BC: black carbon
IQR: interquartile range
OR: odds ratio
CI: confidence interval
ART: assisted reproductive technology
TAPS: twin anemia-polycythemia sequence
MFPR: multifetal pregnancy reduction
MA: maternal age
GDM: gestational diabetes mellitus
TTTS: twin-twin transfusion syndrome
sFGR: selective fetal growth restriction
GA: gestational age
LMP: last menstrual period
CRL: crown-rump length
AOD: aerosol optical depth
BMI: body mass index.
